# Impact of posterior femoral condylar cartilage and posterior intercondylar distance on rotation of femoral component in total knee arthroplasty

**DOI:** 10.1186/s12891-020-03537-2

**Published:** 2020-07-28

**Authors:** Teruyuki Miyasaka, Mitsuru Saito, Daisaburo Kurosaka, Ryo Ikeda, Shoki Yamanaka, Keishi Marumo

**Affiliations:** 1 Department of Orthopaedic Surgery, Toshima Hospital, 33-1 Sakaecho, Itabashi-ku, Tokyo, 173-0015 Japan; 2 Department of Orthopaedic Surgery, Jikei University School of Medicine, 3-25-8 Nishi-Shimbashi, Minato-ku, Tokyo, 105-8461 Japan

**Keywords:** Femoral component, Rotational alignment, Articular cartilage, Total knee arthroplasty, Radiographs, Posterior condyle, Posterior intercondylar distance

## Abstract

**Background:**

Greater accuracy is needed when determining the final femoral component (FC) rotation during total knee arthroplasty (TKA), because this parameter affects soft tissue balance during flexion and patellar tracking. Anatomical markers, such as the epicondylar axis, are typically used to determine the final FC rotation, although intraoperative confirmation may be challenging. Therefore, rotational position is frequently determined with the posterior condylar axis (PCA) as a landmark. However, the thickness of the posterior condylar cartilage has not been considered and may not be represented on preoperative images. We used plain X-rays to measure the thickness of the medial and lateral posterior condylar cartilage fragments postoperatively, and investigated the effects of differences in cartilage thickness on final FC rotation.

**Methods:**

Fifty knees (19 men, 31 women) underwent primary TKA to treat medial knee osteoarthritis at our hospital between August 2015 and May 2017. All knees were treated using an Attune PS (DePuy Synthes, Inc., Warsaw, IN). We first measured the distance between the posterior femoral condyles, resected the posterior condyle, and measured the thickness of the resected cartilage fragments. We then took X-ray images from a direction tangential to the osteotomy surface, secured the cartilage fragments with digital calipers, and measured the thickness of the cartilage. We investigated the effects of differences in cartilage thickness on final FC rotation of the residual medial and lateral cartilage with a trigonometric function.

**Results:**

Medial condylar cartilage thickness averaged 0.6 ± 0.5 mm and the lateral condylar thickness averaged 1.8 ± 0.6 mm; posterior intercondylar distance averaged 46.1 ± 3.3 mm and average impact on rotation of the cartilage remnant was 1.5 ± 0.9° (− 0.1–3.9°). There may be measurement error of up to 4° in the maximum values compared with the preoperative plan in cases with short intercondylar distance.

**Conclusions:**

In cases where the FC external rotation angle is determined using the posterior condyles as landmarks, this angle can be affected by the intercondylar distance, especially in Japanese women who have small physical stature. This angle can potentially be much larger, so caution is advised. Our results suggest that several anatomical landmarks should be referenced to achieve accurate FC rotation.

## Background

High accuracy is required in performing total knee arthroplasty (TKA) in order to determine the rotational positioning of the femoral component (FC) because it can influence patellar tracking and symmetry of the flexion gap [1–4]. Navigation systems and Patient-Specific Instruments (PSI) have been reported to have relatively good performance, but there have been unfavorable reports with respect to FC rotation [5–9].

The transepicondylar axis or Whiteside’s line is often referenced as an anatomical landmark, but verification may be challenging intraoperatively. Therefore, most TKA systems use the posterior condylar axis (PCA) as a landmark intraoperatively for sizing and rotation alignment. However, the cartilage remnant on the posterior condyles varies in thickness medially and laterally and impacts the rotational angle, and is possibly not reflective of preoperative planning estimates. Past reports have described using imaging technology such as computed tomography (CT) arthrograms and magnetic resonance imaging (MRI), and others have documented determining the impacted angle using trigonometry by measuring the cartilage remnant thickness by actually cutting the cartilage remnant on the posterior condyles with a scalpel after making the bone cuts [10–13].

The impact of the posterior femoral condylar cartilage on FC rotation in TKA is defined by the difference in residual cartilage thickness between the posterior femoral condyle and posterior condylar distance. Therefore, we hypothesized that the posterior condylar distance has a greater influence on the rotational position of FC. If this hypothesis is correct, rotational errors of the FC are likely to occur during surgery in patients with small physique.

To clarify this, we obtained plain X-ray images of the cut posterior condyles parallel to the cut surface and measured the cartilage thickness of the intraoperatively excised medial and lateral posterior condyle bone-cartilage fragments by utilizing the differences in radiolucency between cartilage and bone. Then, after making the distal femur bone cuts, we measured the distance between the tips of the posterior condyles for each case and used trigonometry to determine the impact on FC rotational position by differences in cartilage remnant thickness.

## Methods

This study was approved by the institutional review board of our institution. From August 2015 to May 2017, a total of 50 knees (19 men, 31 women) at our institution that underwent primary TKA for medial compartment knee osteoarthritis (OA). Average age was 74.9 ± 7.6 years, average body mass index was 26.8 ± 3.8, and average preoperative hip-knee-ankle angle was 192.2 ± 6.0°. The characteristics of the patients are summarized in Table 1. A single surgeon performed the surgeries, bone cuts were made using CT-free navigation system (BrainLAB AG, Munich, Germany) for the distal femur and proximal tibia. The Attune® PS knee system (DePuy Synthes, Inc., Warsaw, IN) was used in all cases.

**Table 1:** Patient characteristics

	Total (*n* = 50)	Men (*n* = 19)	Women (*n* = 31)	*p*-value Men vs Women
Age (years)	74.9 ± 7.6	72.0 ± 8.8	76.7 ± 6.3	0.03
Height (cm)	153.5 ± 8.7	161.2 ± 7.4	148.9 ± 5.7	< 0.001
Weight (kg)	63.5 ± 12.1	71.9 ± 12.0	58.4 ± 9.0	< 0.001
BMI	26.8 ± 3.8	27.6 ± 3.7	26.3 ± 3.8	0.27
HKA angle (deg)	192.2 ± 6.0	193.4 ± 5.2	191.5 ± 6.4	0.28

*BMI* body mass index, *HKA angle* hip-knee-ankle angle. Values are reported as the mean ± standard deviation

**Fig. 1:**
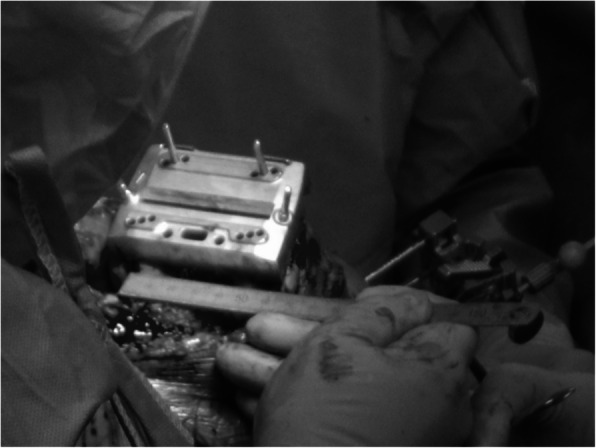
Measuring posterior femoral intercondylar distance (d)

At the start of the operation, a reference frame was attached to the distal femur or the proximal tibia with a bicortical pin. This was followed by surface matching, where the surgeon digitized freely chosen points on the bone surface of both the femur and the tibia. Femoral and tibia cutting blocks were orientated under real-time visualization on the navigation system display. Instruments using standard posterior condyles as landmarks were used for the posterior condyles. For the proximal tibia resection plane, the resection level was set to 10 mm from the deepest point of the higher tibia plateau level. Rotational alignment of the tibial tray was achieved by orientating the tray with reference to the medial third of the tibial tuberosity. After resection, all planes were checked using the verification tool of the navigation system. Intraoperatively, after making bone cuts in the distal femur using a CT-free navigation, the posterior femoral intercondylar distance (d) was measured prior to cutting the posterior condyles. The posterior femoral intercondylar distance (d) was defined as the distance between the contact points of the medial and lateral posterior condyles on the rotational alignment jig. The contact points were marked before measuring the distance (Fig. 1). The measurement was taken once during the operation, because if 2 measurements were taken in series, the possibility of the first measurement influencing the second measurement was likely unavoidable. Instead, we (the lead and assistant surgeons) made sure that the ends of the tape measure were accurately placed on the contact points before carefully taking the measurements.

**Fig. 2:**
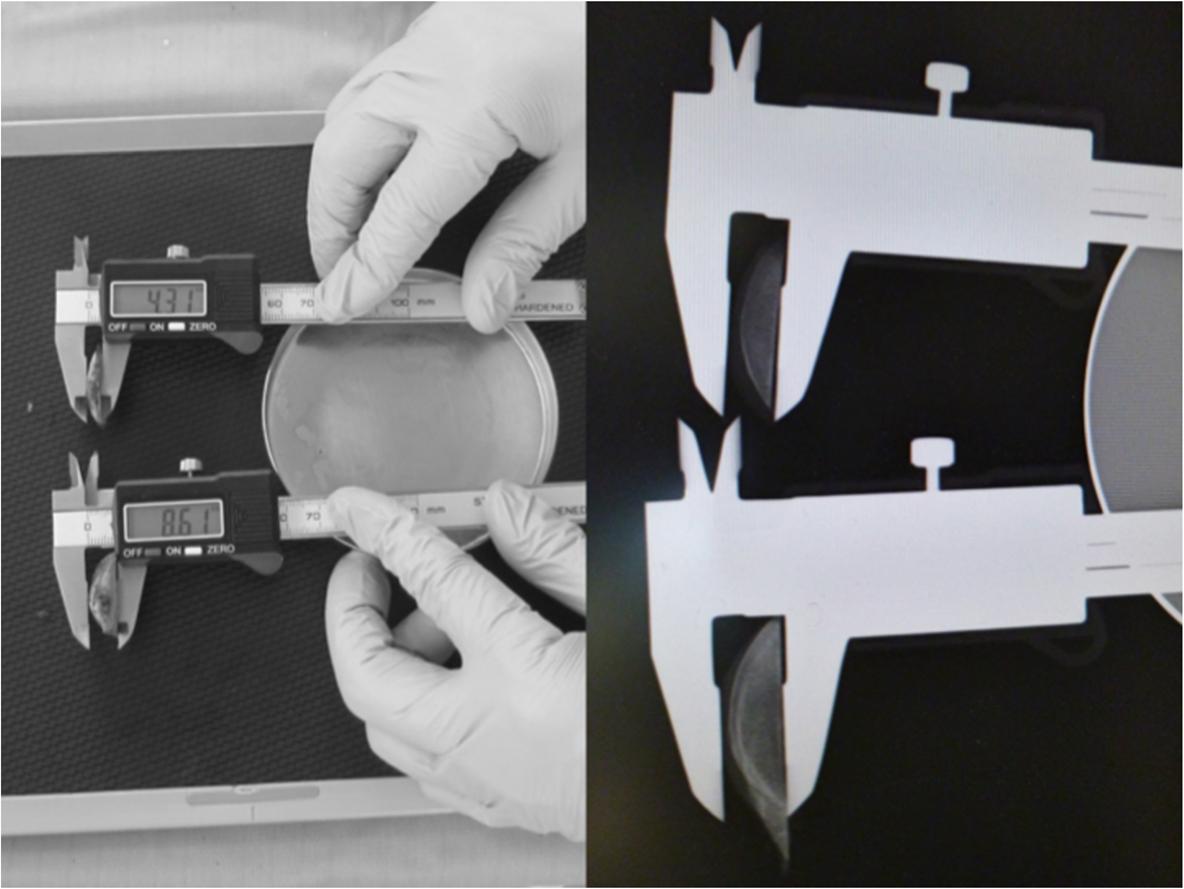
Radiographs taken parallel to the cut surface

**Fig. 3:**
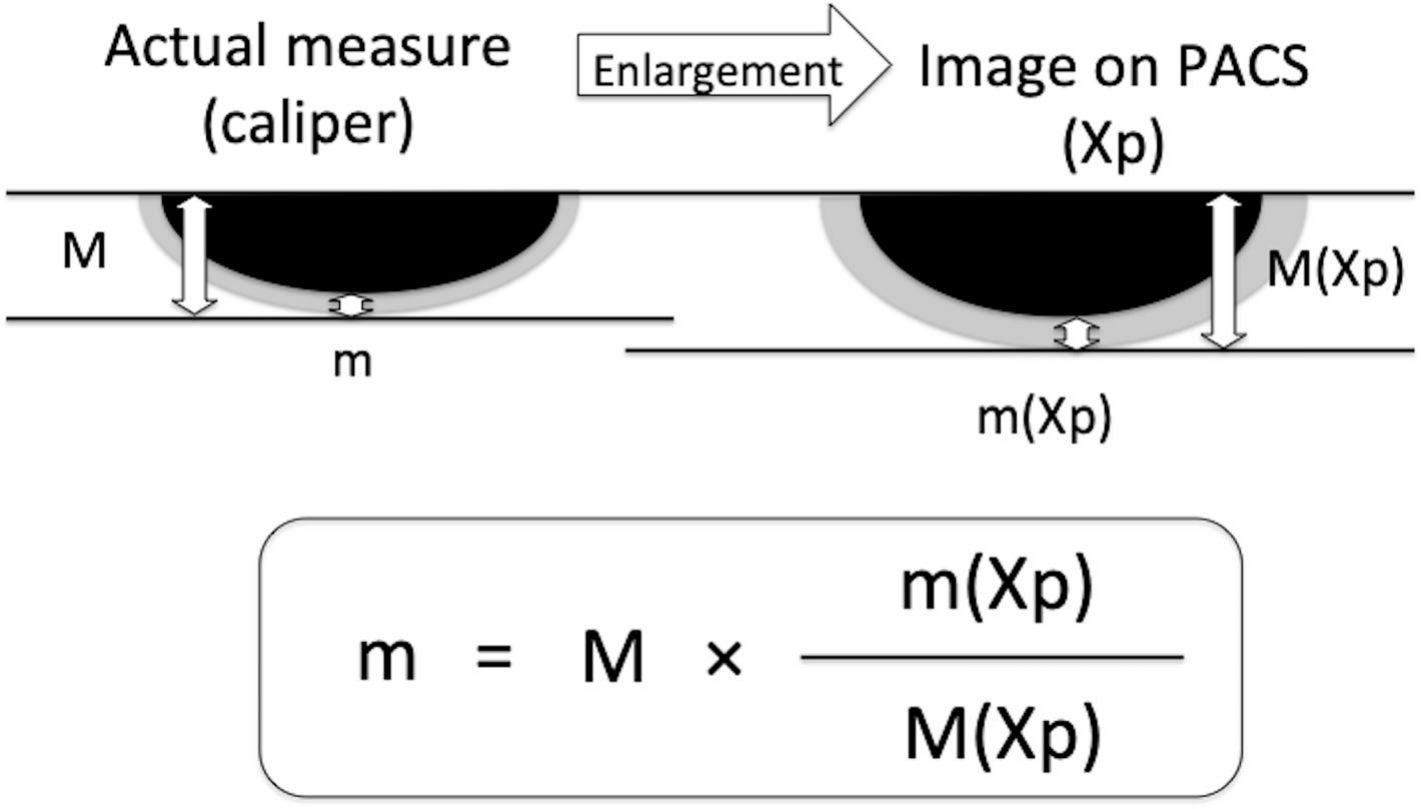
Measuring cartilage thickness (medial side: m; lateral side: l)

**Fig. 4:**
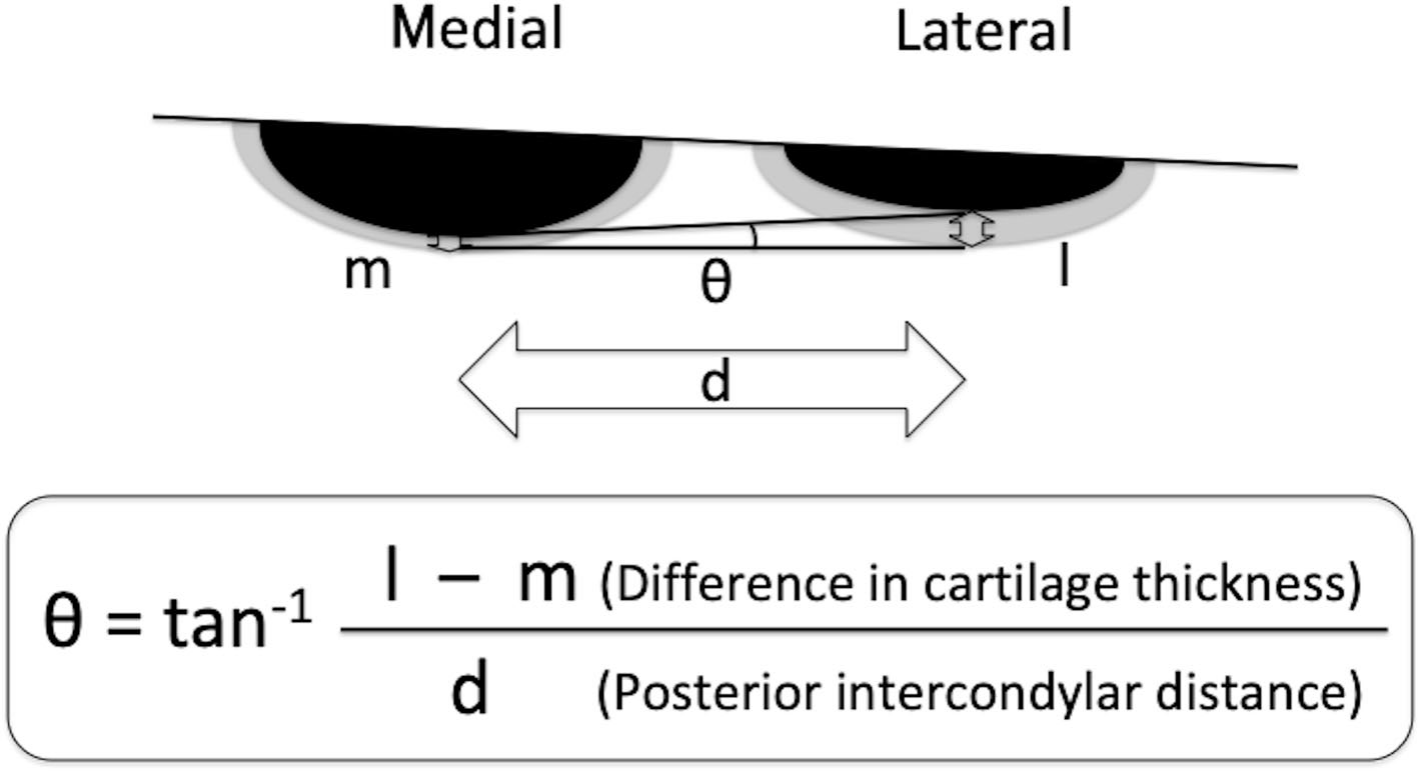
Calculation method for impact on rotation (θ)

Postoperatively, first a film was placed on a stable table, the surface of which was parallel to the ground. The thickest part of the cartilage fragment was secured with a digital caliper, and the position of the fragment was adjusted by checking it from all directions so that the cut surface was perpendicular to the digital caliper. A stable flat petri dish was placed on the film, and then the main scale of the digital caliper was firmly placed on the petri dish so that the cut surface was stably positioned perpendicular to the film. The cut surface was placed directly under the beam head, and the sample was irradiated perpendicular to the ground. After the X-rays were taken, we confirmed whether the cut surface was in a straight line on the PACS images (Fig. 2). The actual thickness of cartilage on a picture archiving and communication system were calculated, even though the acquired images were enlarged compared with actual objects, because the ratio of thicknesses of bone and cartilage would not change with enlargement (Fig. 3). The impact of differences in cartilage remnant thicknesses on the medial and lateral sides (l − m) on rotational position of FC (*θ*) was calculated using trigonometry according to the methods described by Fujii et al. [11] (Fig. 4).

Two observers repeated the measurements twice, and the mean of the four values was regarded as the true value. One observer repeated the measurements 10 times in 3 subjects and assessed the intraobserver variations of the measurements. The maximum intraobserver difference in the measurements was 0.08 mm in cartilage thickness (the largest SD was 0.02) and 0.11 mm in the differences in cartilage remnant thicknesses on the medial and lateral sides (the largest SD was 0.03). Two observers repeated the measurements 10 times in 1 subject and assessed the interobserver variations of the measurements. The maximum interobserver difference was 0.14 mm in the cartilage thickness (the largest SD was 0.04). In theory, the rotation angle reaches a maximum when the posterior intercondylar distance is at a minimum and the difference between the measurements reaches a maximum. When the posterior intercondylar distance is a minimum of 39 mm, the rotation angle is 0.16° because the maximum intraobserver difference in the measurements was 0.08 mm in cartilage thickness. This is 0.21° because the maximum interobserver difference was 0.14 mm in terms of cartilage thickness.

Statistical differences between the sexes were assessed using the Mann–Whitney U test. A *p*-value of < 0.05 was considered to indicate statistical significance.

## Results

**Fig. 5:**
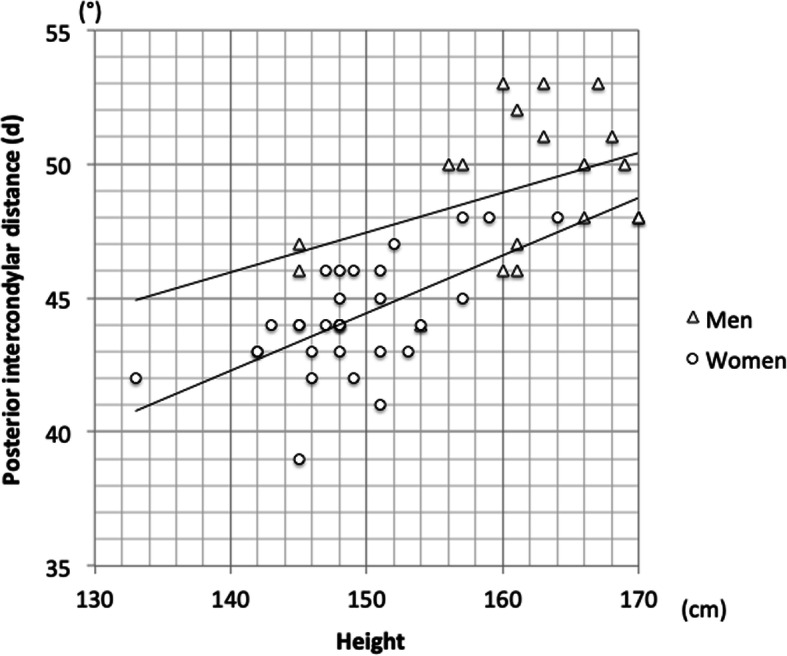
Association between height and posterior intercondylar distance Men: r = 0.404, *p* = 0.086; women: r = 0.601, *p* < 0.001 (Pearson’s correlation coefficient)

**Fig. 6:**
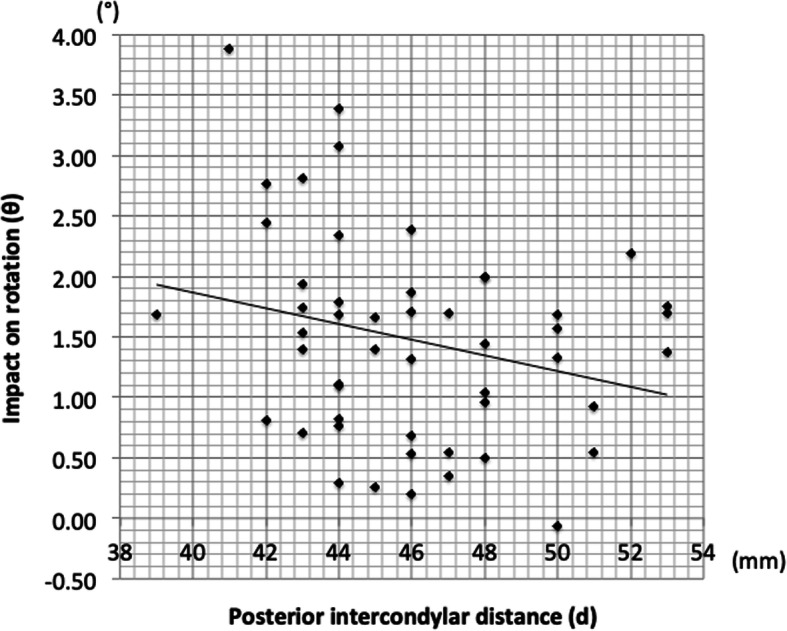
Association between posterior intercondylar distance (d) and impact on rotation (θ). All subjects: r = − 0.219, *p* = 0.126; men: *r* = 0.267, *p* = 0.269; women: *r* = − 0.299, *p* < 0.103 (Pearson’s correlation coefficient)

The mean medial condylar cartilage thickness (m) was 0.9 ± 0.6 mm in men and 0.4 ± 0.4 mm in women; women averaged 0.5 mm less than men (*p* < 0.001). The mean lateral condylar thickness (l) and differences in cartilage thickness between the medial and lateral condylar cartilages (l − m) averaged 0.2–0.3 mm less than that in men but was not significantly different between women and men (*p* = 0.16). The mean posterior intercondylar distance (d) was 46.1 ± 3.3 (39.0–53.0) mm; the maximum difference was 14 mm. Posterior intercondylar distance averaged 49.1 ± 2.7 (44.0–53.0) mm in men and 44.2 ± 2.1 (39.0–48.0) mm in women, and was about 5 mm smaller in women than in men (*p* < 0.001). The average impact on rotation of the cartilage remnant was 1.5 ± 0.9°; the maximum value was 3.9° with no significant different between women and men (*p* = 0.12) (Tables 2 and 3). Regarding height and posterior intercondylar distance, there was a positive correlation between them both in men and in women, and the correlation was significant in women (*p* < 0.001). Women tended to have smaller intercondylar distance for a given height (Fig. 5). Further, the mean posterior intercondylar distance adjusted for the height was 47.7 ± 0.4 mm in men and 45.0 ± 0.4 mm in women, indicating a significant difference (*p* = 0.002, ANCOVA with height as the covariate). Regarding the impact on rotation and posterior intercondylar distance, Pearson’s product-moment correlation analysis confirmed a negative correlation, albeit not significant, in all subjects and in women (Fig. 6). Also, when the rotation angles were compared between the subgroups divided according to the median value, there was a correlation, albeit not significant, in women (*p* = 0.06, Tables 4, 5 and 6). Taken together, in women, for subjects with a small intercondylar distance, the impact on rotation of the cartilage remnant tended to be large.

**Table 2:** Comparison of cartilage thickness, posterior intercondylar distance and impact on rotation between men and women

	Total (*n* = 50)	Men (*n* = 19)	Women (*n* = 31)	*p*-value Men vs Women
Medial side: m (mm)	0.6 ± 0.5 (0.0**–**2.1)	0.9 ± 0.6 (0.3**–**2.1)	0.4 ± 0.4 (0.0**–**1.7)	< 0.001*
Lateral side: l (mm)	1.8 ± 0.6 (0.4**–**3.0)	1.9 ± 0.4 (0.9**–**2.5)	1.7 ± 0.6 (0.4**–**3.0)	0.16
Differences: l − m (mm)	1.2 ± 0.7 (− 0.1**–**2.8)	1.0 ± 0.6 (− 0.1**–**2.0)	1.3 ± 0.7 (0.2**–**2.8)	0.16
Posterior intercondylar distance: d (mm)	46.1 ± 3.3 (39.0**–**53.0)	49.1 ± 2.7 (44.0**–**53.0)	44.2 ± 2.0 (39.0**–**48.0)	< 0.001*
Impact on rotation: θ (°)	1.5 ± 0.9 (− 0.1**–**3.9)	1.2 ± 0.7 (− 0.1**–**2.4)	1.7 ± 0.9 (0.3**–**3.9)	0.12

Data are presented as the mean ± standard deviation. Medial side, m; lateral side, l; differences, l – m; posterior intercondylar distance, d; impact on rotation, θ. *Significant value

**Table 3:** Results from the present and previous studies

Author/ Journal	Methodology	Medial side: m (mm)	Lateral side: l (mm)	Posterior intercondylar distance: d (mm)	Impact on rotation: θ (°)
Asada et al. The Knee (2012)	CT Arthrography				1.1 ± 0.7 (−0.3**–**2.1)
Tashiro et al. KSSTA (2012)	MRI				1.7 ± 0.7 1.5 ± 0.7
Fujii et al. Surg Radiol Anat (2012)	Cartilage excision by scalpel	0.7 ± 0.7 (0.0**–**3.3)	2.1 ± 0.7 (0.0**–**3.8)		1.7 ± 1.3 (0.0**–**4.6)
Hamada et al. J Med Invest (2017)	MPR imaging and navigation				1.7 ± 1.2
**Present study**	**Radiograph**	**0.6 ± 0.5 (0.0–2.1)**	**1.8 ± 0.6 (0.4–3.0)**	**46.1 ± 3.3 (39.0–53.0)**	**1.5 ± 0.9 (**−**0.1–3.9)**

*CT* computed tomography, *MPR* multiplanar reformation, *MRI* magnetic resonance imaging; Data are presented as the mean ± standard deviation

**Table 4:** Comparison of external rotation angle in all subjects by subgroups divided according to median value

	Posterior intercondylar distance (subgrouping according to the median value)				*p*-value
	Short distance group (< 46 mm)		Long distance group (≥46 mm)		
	n	Data	n	Data	
Angle	24	1.647 ± 0.949	26	1.258 ± 0.720	0.113

Data are presented as the mean ± standard deviation. *p*-value: Welch’s t-test

**Table 5:** Comparison of external rotation angle between subgroups of men divided according to median value

	Posterior intercondylar distance (subgrouping according to the median value)				*p*-value
	Short distance group (≤51 mm)		Long distance group (> 51 mm)		
	n	Data	n	Data	
Angle	11	0.991 ± 0.783	8	1.457 ± 0.668	0.182

Data are presented as the mean ± standard deviation. p-value: Welch’s t-test

**Table 6:** Comparison of external rotation angle between subgroups of women divided according to median value

	Posterior intercondylar distance (subgrouping using the median as a split point)				*p*-value
	Short distance group (≤44 mm)		Long distance group (> 44 mm)		
	n	Data	n	Data	
Angle	20	1.800 ± 0.949	11	1.243 ± 0.628	0.060

Data are presented as the mean ± standard deviation. *p*-value: Welch’s t-test

## Discussion

In this study, we measured the intercondylar distance and differences in medial and lateral cartilage thickness for each case to investigate how intercondylar distance and cartilage remnants on the posterior condyles impact rotation. The medial remaining cartilage was significantly thinner in women (*p* < 0.001). Hanna et al. [14] reported that women have increased rates of cartilage loss and progression of cartilage defects at the knee than men. Given that subjects of this study were patients with medial osteoarthritis, the remaining medial cartilage appeared to thin particularly in women. For subjects with small intercondylar distance, the impact on rotation of the cartilage remnant tended to be larger and the greatest discrepancy found was 3.9° when compared with the value in preoperative planning.

According to reports from various perspectives [10–13], the impact of cartilage remnants on rotation averages 1.1–1.7°, which is in agreement with the average of 1.5° from the present study (Table 1). Asada et al. [10] studied CT arthrograms of 31 knees and reported that the influence of posterior condyle cartilage on rotation was 1.1° on average and 2.1° at greatest. Tashiro et al. [13] reported this effect using MRI. However, the articular cartilage thickness shown on CT or MRI could vary depending on imaging resolution. Furthermore, in these studies, osteotomy might not have been performed according to preoperative three-dimensional planning or the actual influence on rotation might have not been assessed correctly because the removed osteochondral fragments were not measured directly. Fujii et al. [11] gave no details in their reports of intercondylar distances but did excise cartilage remnants with a scalpel from posterior condyles that were actually cut. They evaluated the differences in thickness before and after excision as the thickness of the cartilage remnant and reported the average as 1.7° and 4.6° at maximum. They also reported the average of the medial condylar cartilage as 0.7 ± 0.7 (0.0–3.3) mm and of the lateral condylar cartilage as 2.1 ± 0.7 (0.0–3.8) mm. Cartilage from our study was smaller, with an average of 0.61 ± 0.54 (0.00–2.05) mm on the medial condyle and 1.78 ± 0.56 (0.39–2.99) mm on the lateral condyle; the greatest value for the medial side was 1.25 mm and for the lateral side was 0.81 mm. This may be due to the accuracy of excising the cartilage with a scalpel or from the process of repositioning the bone-cartilage fragment into a caliper after the cartilage had already been measured with a caliper and was then scraped off. The measuring technique we used is performed by taking a single plain X-ray image of the excised bone fragments, and thus is relatively easy, low cost, and has no radiation exposure.

Varying the distal femoral cuts would impact the posterior condyle cuts. Therefore, for the distal femoral cuts, we used a navigation system that is considered to have high accuracy [5–7]. Not only is the affected angle of rotation due to cartilage remnant on posterior condyles influenced by differences in medial and lateral cartilage thickness, but as demonstrated by trigonometric calculations, it is also affected by the intercondylar distance. However, there are no detailed reports on these issues. In the present study, among subjects with small intercondylar distance, the greatest discrepancy was almost 4° compared with the value obtained during preoperative planning. In addition, 8 subjects who had a discrepancy of > 2.4° also had a posterior intercondylar distance averaging 43.3 ± 1.6 (41–46) cm and all were below the average posterior intercondylar distance of 46.1 cm.

Ishimaru et al. [15] reported that Japanese women had a relatively narrower femoral width for a given AP length compared with men. Also, our results reveal that posterior intercondylar distance tended to be smaller in women than in men for a given height. Moreover, there was no significant difference in the cartilage thickness between men and women (Differences: l − m). Thus, in patients with medial compartment OA, if preoperative planning is performed using images where information on cartilage cannot be obtained, or in cases where the external rotation angle of the FC is determined using the posterior condyles as landmarks, this angle can be affected by the intercondylar distance. This is especially the case in Japanese women who have small physical stature, and this angle can potentially be much larger and therefore caution is necessary.

Lee et al. [16] reported that rotational femoral and tibial articulation mismatch can lead to patellofemoral dysfunction, knee instability, and tibial polyethylene insert wear. Internal rotation of the femoral component moves the groove portion of the femoral component relatively medially, and increases the lateral force vector on the patella [17]. External malrotation of the femoral components could theoretically worsen tibiofemoral congruity and increase the risk of polyethylene insert wear over the long-term, causing residual pain after TKA. Tsukiyama et al. [18] showed that knees with postoperative medial joint laxity > 3° in flexion resulted in inferior patient satisfaction and knee function as evaluated by the 2011 Knee Society Knee Scoring System. Luyckx et al. [19] reported that posterior translation of the lateral condyle and relative internal tibial rotation during flexion forced stretching or friction of the iliotibial band and caused lateral knee pain in 77 cases (7.2%) in a series of 1070 TKAs.

Our study has some limitations. First is the fact that cases are limited to Japanese patients. Because posterior intercondylar distance is predicted to be larger in Western patients than in Asian patients, the impact of cartilage on rotation may likely be smaller on average. Second, all had varus knees and no valgus knees were included, because osteoarthritic valgus knee is rare in Japan. Third, the accuracy of the measurements of the distance between the medial and lateral posterior condyle is possibly not high.

## Conclusions

In cases where the FC external rotation angle is determined using the posterior condyles as landmarks, this angle can be affected by the intercondylar distance. This is especially the case in Japanese women who have small physical stature, and this angle can potentially be much larger, and therefore caution is necessary. Our results seem to indicate that several anatomical landmarks should be referenced in order to achieve accurate rotation of the FC.

## Data Availability

The datasets used and/or analyzed during the current study are available from the corresponding author on reasonable request.

## Abbreviations

BMI: Body mass index
CT: Computed tomography
FC: Femoral component
HKA angle: Hip-knee-ankle angle
MPR: Multiplanar reformation
MRI: Magnetic resonance imaging
OA: Osteoarthritis
PCA: Posterior condylar axis
PSI: Patient Specific Instruments
TKA: Total knee arthroplasty
